# Insights into female sperm storage from the spermathecal fluid proteome of the honeybee *Apis mellifera*

**DOI:** 10.1186/gb-2009-10-6-r67

**Published:** 2009-06-18

**Authors:** Boris Baer, Holger Eubel, Nicolas L Taylor, Nicholas O'Toole, A Harvey Millar

**Affiliations:** 1 ARC Centre of Excellence in Plant Energy Biology, The University of Western Australia, Stirling Hwy, Crawley WA 6009, Australia; 2 Centre for Evolutionary Biology, School of Animal Biology, The University of Western Australia, Stirling Hwy, Crawley WA 6009, Australia; 3 Centre of Excellence for Computational Systems Biology, The University of Western Australia, Stirling Hwy, Crawley WA 6009, Australia

## Abstract

**Background:**

Female animals are often able to store sperm inside their body - in some species even for several decades. The molecular basis of how females keep non-own cells alive is largely unknown, but since sperm cells are reported to be transcriptionally silenced and, therefore, limited in their ability to maintain their own function, it is likely that females actively participate in sperm maintenance. Because female contributions are likely to be of central importance for sperm survival, molecular insights into the process offer opportunities to observe mechanisms through which females manipulate sperm.

**Results:**

We used the honeybee, *Apis mellifera*, in which queens are highly polyandrous and able to maintain sperm viable for several years. We identified over a hundred proteins representing the major constituents of the spermathecal fluid, which females contribute to sperm in storage. We found that the gel profile of proteins from spermathecal fluid is very similar to the secretions of the spermathecal gland and concluded that the spermathecal glands are the main contributors to the spermathecal fluid proteome. A detailed analysis of the spermathecal fluid proteins indicate that they fall into a range of different functional groups, most notably enzymes of energy metabolism and antioxidant defense. A metabolic network analysis comparing the proteins detected in seminal fluid and spermathecal fluid showed a more integrated network is present in the spermathecal fluid that could facilitate long-term storage of sperm.

**Conclusions:**

We present a large-scale identification of proteins in the spermathecal fluid of honeybee queens and provide insights into the molecular regulation of female sperm storage.

## Abstract 2

A proteomic and metabolic network analysis of honeybee queen spermathecal fluid provides insights into female long-term sperm storage mechanisms.

## Background

Sperm storage by females is widespread throughout the animal kingdom [1,2] but amazingly little is known about how females are able to keep sperm cells viable over prolonged periods of time. In many species, females provide specialized morphological structures for sperm storage often known as spermathecae [3]. Females 'interact' with and 'sustain' sperm that are stored in these structures through glandular secretions, produced, for example, by the spermathecal glands [4]. These secretions contain proteins, metabolites and other chemicals in the honeybee *Apis mellifera *[5] and spermathecal fluid has recently been shown to maintain sperm viability [6,7]. Several proteins have been proposed to be responsible for this effect, such as the glycolytic enzyme triosphosphate isomerase [5] and a number of antioxidant defense enzymes [8]. In addition, high K^+ ^concentrations and the high pH of the spermathecal fluid have been proposed to lower the metabolic rate of sperm in storage [5,9,10]. However, despite the spermatheca containing 5 to 10 mg of protein/ml [5], no systematic analysis of these female derived proteins has so far been conducted. As a consequence, our knowledge about the biochemical and physiological mechanisms that maintain sperm viability or the physiological costs associated with sperm storage are extremely limited [11]. Furthermore, females have been hypothesized to bias paternity outcomes by manipulating sperm in storage [12]. Consequently, sexual selection [13] may influence the female contributions towards stored sperm as well.

The study of male contributions towards sperm, such as seminal fluids or male accessory gland secretions, has received much more attention [14-16]. Males transfer a complex mixture of components to the female along with sperm [13,17-21], which have multiple effects on sperm viability or female physiology [6,7] but some of these components also seem to be agents of sexual conflict [22-25]. It seems reasonable to assume that females have also evolved a complementary arsenal of components to support and manipulate sperm. This makes detailed studies of female sperm storage physiology and its interactions with sperm and/or seminal fluid timely. A crucial step to understand female influence on stored sperm is to identify the components provided by the female, and proteomic technologies offer the opportunity to investigate the female's arsenal.

Social hymenopteran insects (the bees, ants and wasps) are interesting model systems to study sperm storage by females because several species have taken sperm storage to spectacular extremes [11,26,27]. This can be seen in terms of both the total number of sperm stored as well as the efficiency by which sperm are kept alive over prolonged periods of time [28]. A phenomenon common to many social hymenopteran insects is that queens only copulate during a brief period early in life [16,29,30]. In the absence of re-mating later in life, queens acquire and store a lifetime supply of sperm that often fixes the upper limit of a colony's size, longevity and fitness. Apart from the total number of initially stored sperm, queen lifetime fecundity is also influenced by her efficiency to keep sperm viable. Some social insect queens can not only live for several decades [26,31], but they also maintain colonies of several million workers [11,30,32]. Selection is therefore expected to have maximized storage efficiency of sperm number [28] and sperm survival and minimized sperm number used per egg fertilization. Sperm storage induces costs for the female that are known to trade off with other female life history traits in leaf cutter ants [11] and bumblebees [33]. Finally, in polyandrous species, ejaculates of several males can coexist within the spermatheca for years, but it remains to be investigated whether sperm competition or cryptic female choice occurs whilst sperm is in storage [29].

We have used the honeybee, *A. mellifera*, and present a proteomic identification of the female's contribution towards sperm by identifying proteins that females provide to sperm in storage. Honeybee queens are efficient sperm storers that initially store around 6 million sperm for up to 7 years, giving them an estimated potential to sire up to 1.7 million offspring (see [29] for a review on the honeybee mating system). Consequently, spermathecal fluid components are expected to maximize the survival of large numbers of sperm. Furthermore, honeybee queens are highly polyandrous and store sperm from several males. Consequently, females could use sperm storage to manipulate sperm and, thus, manipulate paternity success. An additional advantage of honeybees as a model system is that the availability of the honeybee genome sequence [34] allows the use of tandem mass spectrometry (MS/MS) to identify proteins [19,20,35]. We here identify the spermathecal fluid proteome of honeybee and compare it to recently published proteomic profiles of sperm and seminal fluid [19,20] in order to understand the specific female contribution to sperm in storage.

## Results

**Figure 1:**
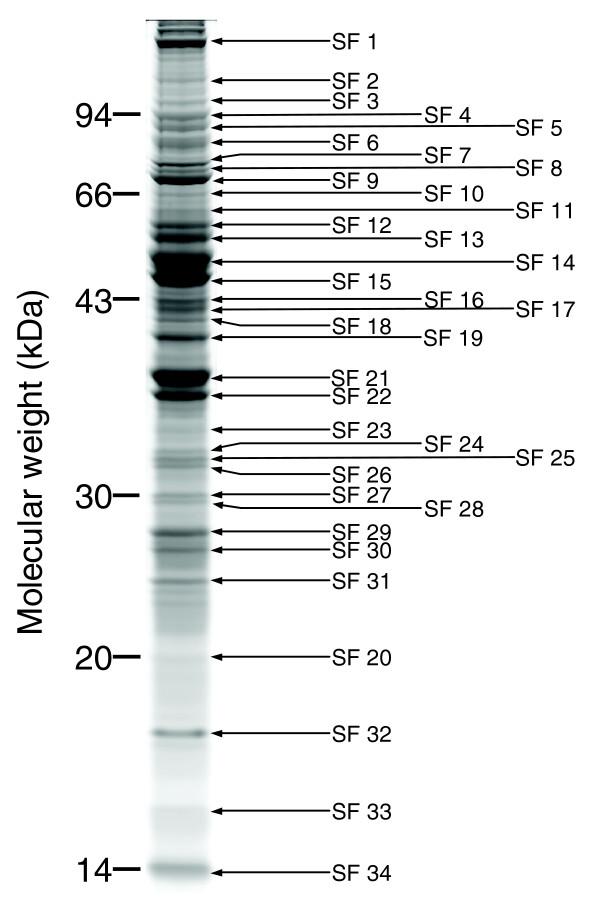
SDS-PAGE gel separation of spermathecal fluid proteins. A colloidal Coomassie blue stained gel showing a representative protein profile of spermathecal fluid. A total of 50 μl of spermathecal fluid (SF) extract was loaded on the gel. Thirty-four protein bands, as indicated by arrows, were excised for protein identification. An overview of significant protein identifications for these bands is given in Additional data file 1.

**Figure 2:**
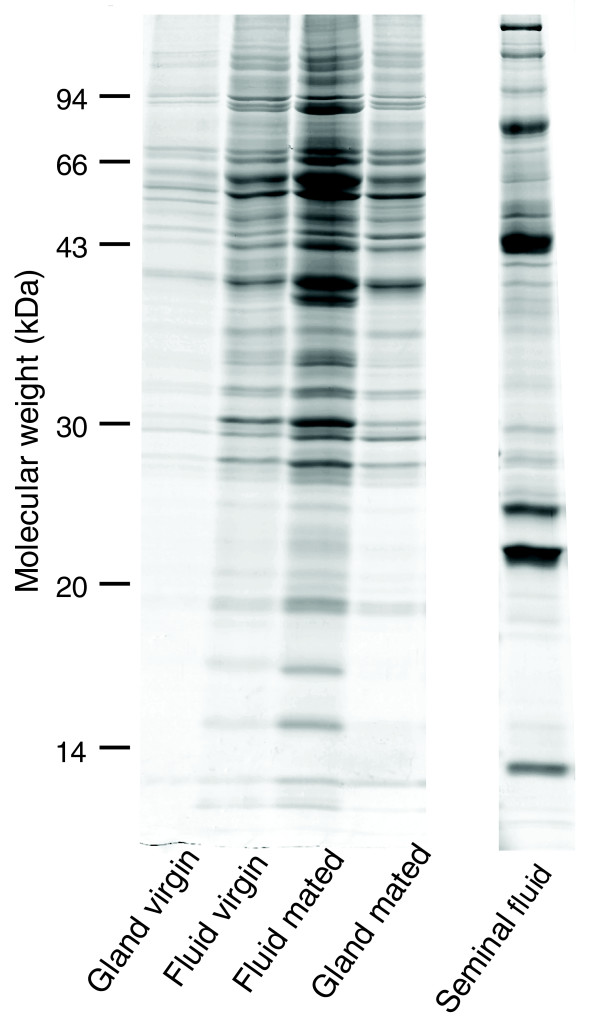
Spermathecal gland and spermathecal fluid proteins in mated and virgin queens. Colloidal Coomassie blue stained gel lanes showing representative protein profiles of spermathecal fluid and spermathecal gland secretions from virgin and mated queens and seminal fluid. A total of 8 μl of fluid extracts from the spermathecal samples and 16 μl of the seminal fluid sample were loaded on the gels.

The proteins of spermathecal fluid collected from dissected spermathecae were separated by one-dimensional SDS-PAGE (Figure 1). We compared this profile to extracted spermathecal wall proteins, hemolymph and sperm. In each case the protein profiles were distinct, showing that separation of these protein subsets could be achieved by our dissection and extraction protocols ([19] and data not shown). Protein profiles of spermathecal fluid were visually inspected on a total of 11 one-dimensional gels using 12 independent biological replicates for mated and 4 independent biological replicates for virgin queens. We found that specific protein profiles for spermathecal fluid can be consistently reproduced (Figure 1), in both technical and biological replicates and resemble those found in earlier studies [5,7]. Modifications of our standardized extraction protocol resulted in no obvious abundance changes of protein profiles on the gels, indicating that our collection method is a reliable way to sample spermathecal fluid. We found a large overlap in the spermathecal fluid protein band profiles of mated and virgin queens (Figure 2). Furthermore, the protein profile of the spermathecal gland secretions is very similar to that of the spermathecal fluid, both for mated and virgin queens (Figure 2). The protein profiles of spermathecal fluid were very different from that of seminal fluid isolated from male ejaculates (Figure 2).

To identify the most abundant proteins present in the spermathecal fluid, we ran a total of four mass spectrometry analyses from four independent biological samples. Two sets of analyses were performed, one based on in-gel digested bands of one-dimensional SDS-PAGE (Figure 1) and a second based on liquid chromatography (LC)-MS/MS analysis of total protein tryptic digests. The latter were nested experiments each consisting of six LC-MS/MS experiments performed in series, with the peptides identified in each run excluded from the subsequent analysis to improve the depth of analysis (see Materials and methods).

A summary of all significant protein identifications is given in Table 1 (protein match data are presented in Additional data file 1). Our final analysis resulted in the identification of 122 different proteins across the four spermathecal fluid samples. This set of proteins included molecular chaperones, an array of enzymes involved in energy and amino acid metabolism, antioxidant enzymes, proteins involved in signaling pathways, structural proteins, and a range of proteins with unknown functions (Table 2).

**Table 1:** Proteins in honeybee queen spermathecal fluid

	Mated		Virgin				
						
PreRelease2 accession	Gel	LC MS/MS	LC MS/MS	Seminal fluid	Sperm	Pred. secret.	Protein functional description
GB10467-PA	X	X		X			Aspartate aminotransferase 2 precursor
GB10973-PA		X	X	X			Arginine kinase
GB15049-PA			X				Delta-1-pyrroline-5-carboxylate synthetase
GB15171-PA	X	X			X		Ornithine aminotransferase precursor
GB16218-PA			X				Proline oxidase
GB17641-PA	X	X	X				Alanine aminotransferase 2
GB18844-PA	X	X	X				Glutamate oxaloacetate transaminase 1
GB10133-PA	X	X					Superoxide dismutase
GB10498-PA	X	X	X				Peroxiredoxin
GB12029-PA	X						Glyoxalase domain-containing protein
GB14972-PA	X					S	Thioredoxin reductase
GB15855-PA	X						Thioredoxin-2
GB18955-PA		X	X	X		S	Phospholipid hydroperoxide glutathione peroxidase
GB19380-PA		X	X				Thioredoxin peroxidase 1
GB30268-PA	X	X	X				Glutathione s transferase S1
GB12586-PA			X			S	Protein disulfide-isomerase precursor
GB12447-PA	X						CAP, adenylate cyclase-associated protein 1
GB13596-PA			X				ATP synthase
GB14791-PA			X				ATP synthase subunit
GB15291-PA			X				ATP synthase gamma subunit
GB16485-PA			X				ATP synthase D chain
GB10989-PA	X	X	X				Vacuolar ATPase catalytic subunit A
GB11380-PA	X	X	X				Vacuolar H+ ATPase 44 kDa C subunit
GB12913-PA	X	X	X				Vacuolar proton pump E subunit
GB13499-PA	X	X	X				Vacuolar ATPase subunit G
GB15226-PA	X	X					Vacuolar ATPase subunit D 1
GB17480-PA	X	X	X				Vacuolar ATPase subunit H
GB17499-PA			X				ADP/ATP translocase
GB19171-PA	X	X	X				Vacuolar ATPase 55 kDa B subunit
GB20017-PA	X						Endoplasmic reticulum ATPase
GB10355-PA		X	X			S	Melittin
GB10695-PA	X	X	X		X		Pyruvate kinase
GB10992-PA	X						ATP citrate lyase isoform A
GB11056-PA	X	X	X	X			Phosphoglycerate kinase isoform 1
GB11461-PA	X						UTP-glucose-1-phosphate uridylyltransferase
GB12488-PA			X				Aconitase
GB12573-PA		X	X	X			Citrate synthase
GB12741-PA			X				Aldehyde dehydrogenase
GB12949-PA		X	X				6-Phosphogluconate dehydrogenase
GB13058-PA		X			X		Dihydroxyacetone kinase 2
GB13237-PA	X	X	X				Phosphogluconate mutase
GB13882-PA	X	X					L-lactate dehydrogenase
GB13955-PA		X	X				N-acetyltransferase 5
GB14517-PA		X	X				Isocitrate dehydrogenase
GB14798-PA	X	X	X		X		Glyceraldehyde-3-phosphate dehydrogenase 2
GB14803-PA		X	X			S	Alpha,alpha-trehalase
GB15039-PA	X	X	X				Enolase
GB15052-PA	X	X	X				Phosphoglyceromutase
GB15463-PA		X			X		Aldolase
GB15543-PA		X		X			Malate/L-lactate dehydrogenases
GB15619-PA	X						Transketolase-like
GB15888-PA		X	X				Carbonic anhydrase
GB16429-PA	X	X	X	X			Glucose-6-phosphate isomerase
GB16464-PA	X	X	X				malate dehydrogenase
GB16951-PA	X						Malic enzyme
GB17113-PA	X	X	X				Phosphofructokinase
GB17473-PA	X	X	X	X			Triosephosphate isomerase 1
GB18109-PA		X			X		Aldose reductase (NADP+)
GB18727-PA	X	X	X				Malate dehydrogenase
GB19030-PA	X	X	X				Aldo/keto reductase family protein
GB19387-PA		X	X		X		Hexokinase A, isoform A
GB19460-PA	X	X	X				Aldolase, isoform F
GB11665-PA		X	X	X		S	Chitinase-like protein
GB11876-PA			X			S	LDLa domain containing chitin binding protein
GB16986-PA		X	X			S	Endochitinase precursor
GB10397-PA			X				Alpha-crystallin
GB10800-PA	X						T-complex protein 1
GB10836-PA	X						HSP70
GB14758-PA	X	X	X	X			Heat shock protein 90
GB14852-PA	X	X	X	X			Heat shock protein 8 isoform 1
GB15016-PA	X	X	X				Heat shock protein cognate 3
GB17056-PA	X	X	X				Cyclophilin 1
GB18662-PA			X				Alpha-crystallin, small HSP
GB18969-PA	X		X				Heat shock protein 60
GB12818-PA			X				Histone 2A
GB14548-PA		X	X			S	Deoxyribonuclease II
GB16515-PA	X						ATP dependent DNA helicase
GB19247-PA	X						Elongation factor 2, isoform 1
GB16568-PA			X	X			Cytochrome c oxidase subunit
GB19293-PA	X	X	X				Cytochrome c
GB19729-PA	X	X				S	Cytochrome c
GB11059-PA	X						Retinoid- and fatty-acid binding protein
GB15044-PA		X	X	X			Phosphatidylethanolamine-binding protein
GB14639-PA		X	X			S	Major royal jelly protein 8
GB16324-PA		X	X			S	Major royal jelly protein 9
GB12951-PC	X	X	X				14-3-3-like protein
GB15202-PA		X					DJ-1, neuroprotective transcriptional co-activator
GB15582-PA		X	X				14-3-3 epsilon
GB16178-PA	X						Neuropeptide Y receptor
GB16716-PA			X			S	Leucine-rich repeat-containing protein
GB16072-PA	X	X	X				Iron regulatory protein 1B
GB10536-PA			X				Odorant binding protein 14
GB19662-PA		X				S	Juvenile hormone binding protein
GB19745-PA		X				S	Transferrin
GB10009-PA	X	X	X		X		Tubulin alpha-1 chain
GB10091-PA			X			S	Cuticlin-1 precursor
GB10122-PA		X	X		X		Tubulin, beta, 2
GB10275-PA	X	X	X	X			Tubulin isoform B
GB10514-PA	X	X	X		X		alpha tubulin
GB11282-PA	X	X	X				Moesin isoform D
GB11920-PA	X						Tubulin
GB12614-PA		X					Actin
GB13049-PA		X					Tubulin, beta, 2
GB13229-PA	X						PDZ and LIM domain protein
GB13999-PA	X	X	X			S	Vitellogenin
GB15794-PA			X			S	Cuticlin-1 precursor
GB16448-PA		X	X	X			Annexin IX
GB17673-PA	X						Talin-1
GB17681-PA	X	X	X	X			Actin-5C isoform 1
GB18917-PA		X	X				Cofilin/actin-depolymerizing factor homolog
GB12113-PA			X				Porin
GB14012-PA			X				Phosphate carrier
GB16577-PA			X				Sialin, inorganic phosphate cotransporter
GB11987-PA		X		X			Unknown
GB12562-PA	X	X	X				Hypothetical protein
GB13778-PA	X					S	Unknown
GB14970-PA	X						Muscle-specific protein 300
GB15662-PA	X	X	X	X			Unknown
GB17311-PA		X	X	X		S	Unknown
GB17500-PA			X			S	Hypothetical protein
GB19255-PA	X					S	Osiris 14 CG1155-PA
GB30569-PA		X	X			S	Hypothetical protein

Proteins were identified by MS/MS analysis of one-dimensional gel and gel-free analysis of tryptic peptides from spermathecal fluid extracted from mated and virgin queens. Data are compared to the previous reported analysis of the male seminal fluid and sperm proteomes [19]. Details of the mass spectrometry analysis and detailed matching are shown in Additional data file 1. Significant identification of peptides from a specific bee locus number are shown with an 'X' for gel analyses; the number of proteins predicted to be secreted (Pred. secret.) are shown with an 'S' based on consensus of analysis of amino acid sequences using two different software packages TargetP [52] and IPsort [53].

**Table 2:** Proteins in honeybee queen spermathecal fluid

PreRelease2 accession	RefSeq GI	Bee gene ID	Functional group	Dm match
GB10467-PA	110755553	412675	AA metabolism	CG4233
GB10973-PA	58585146	550932	AA metabolism	CG32031
GB15049-PA	66500225	412948	AA metabolism	CG7470
GB15171-PA	110763628	410582	AA metabolism	CG8782
GB16218-PA	66559229	411808	AA metabolism	CG1417
GB17641-PA	66563168	409196	AA metabolism	CG1640
GB18844-PA	110775909	726943	AA metabolism	CG8430
GB10133-PA	66513527	409398	AntiOx	CG11793
GB10498-PA	66535082	551975	AntiOx	CG11765
GB12029-PA	66517659	552722	AntiOx	CG1532
GB14972-PA	48140590	410032	AntiOx	CG2151
GB15855-PA	48104680	409451	AntiOx	CG31884
GB18955-PA	110756698	726269	AntiOx	CG12013
GB19380-PA	66548188	409954	AntiOx	CG1633
GB30268-PA	66534655		AntiOx	CG8938
GB12586-PA	66531851	551435	AntiOx/Chaperone	CG6988
GB12447-PA	110766149	410158	ATP related	CG5061
GB13596-PA	110762902	551766	ATP synthesis	CG11154
GB14791-PA	48100966	409114	ATP synthesis	CG3612
GB15291-PA	66554156	552699	ATP synthesis	CG7610
GB16485-PA	48098315	410557	ATP synthesis	CG6030
GB10989-PA	66515272	551093	ATP/transport	CG3762
GB11380-PA	110756584	411892	ATP/transport	CG8048
GB12913-PA	66556287	552720	ATP/transport	CG1088
GB13499-PA	66553147	551961	ATP/transport	CG6213
GB15226-PA	66515294	411295	ATP/transport	CG8186
GB17480-PA	-	409055	ATP/transport	CG17332
GB17499-PA	58531215	406075	ATP/transport	CG16944
GB19171-PA	66531434	551721	ATP/transport	CG17369
GB20017-PA	66534286	409377	ATP/transport	CG2331
GB10355-PA	58585154	406130	Bee venom	-
GB10695-PA	66548684	552007	C metabolism	CG7070
GB10992-PA	66530142	550686	C metabolism	CG8322
GB11056-PA	110763826	411576	C metabolism	CG3127
GB11461-PA	66536233	412069	C metabolism	CG4347
GB12488-PA	48098039	408446	C metabolism	CG9244
GB12573-PA	66521738	410059	C metabolism	CG3861
GB12741-PA	66530423	550687	C metabolism	CG3752
GB12949-PA	66547531	552712	C metabolism	CG3724
GB13058-PA	110763782	413697	C metabolism	-
GB13237-PA	66561330	411897	C metabolism	CG5165
GB13882-PA	110758428	411188	C metabolism	CG10160
GB13955-PA	66517612	414027	C metabolism	CG14222
GB14517-PA	110764717	551276	C metabolism	CG7176
GB14798-PA	48142692	410122	C metabolism	CG12055
GB14803-PA	66524360	410484	C metabolism	CG9364
GB15039-PA	110761968	552678	C metabolism	CG17654
GB15052-PA	66550890	552736	C metabolism	CG1721
GB15463-PA	110748959	725455	C metabolism	CG6058
GB15543-PA	66523770	410520	C metabolism	CG10512
GB15619-PA	110751363	550804	C metabolism	CG8036
GB15888-PA	48095863	408827	C metabolism	CG7820
GB16429-PA	66499293	551154	C metabolism	CG8251
GB16464-PA	66513092	408950	C metabolism	CG7998
GB16951-PA	110761561	411813	C metabolism	CG10120
GB17113-PA	-	724724	C metabolism	CG4001
GB17473-PA	148224276	726117	C metabolism	CG2171
GB18109-PA	66525576	551968	C metabolism	CG6084
GB18727-PA	66506786	411014	C metabolism	CG5362
GB19030-PA	110763386	552018	C metabolism	CG10638
GB19387-PA	66525954	551005	C metabolism	CG3001
GB19460-PA	110748949	550785	C metabolism	CG6058
GB11665-PA	66514614	413324	Cell wall degradation, antifungal	CG1780
GB11876-PA	110760993	551323	Cell wall degradation, antifungal	CG8756
GB16986-PA	66511507	551600	Cell wall degradation, antifungal	CG9307
GB10397-PA	110750754	724274	Chaperone	CG4533
GB10800-PA	66563290	409296	Chaperone	CG8977
GB10836-PA	66505007	408706	Chaperone	CG6603
GB14758-PA	110758212	408928	Chaperone	CG1242
GB14852-PA	66537940	409418	Chaperone	CG4264
GB15016-PA	110754998	409587	Chaperone	CG4147
GB17056-PA	66534750	409890	Chaperone	CG9916
GB18662-PA	110750756	410087	Chaperone	CG4533
GB18969-PA	66547450	409384	Chaperone	CG12101
GB12818-PA	110749634	725450	DNA/RNA	CG31618
GB14548-PA	48138800	413489	DNA/RNA	CG7780
GB16515-PA	110768389	412756	DNA/RNA	CG31916
GB19247-PA	66508439	409167	DNA/RNA	CG2238
GB16568-PA	66534766	552610	Electron transport	CG11015
GB19293-PA	48096996	408270	Electron transport	CG17903
GB19729-PA	110760474	724543	Electron transport	CG17903
GB11059-PA	110758758	408961	Lipid metabolism	CG11064
GB15044-PA	66524882	408516	Lipid metabolism	CG6180
GB14639-PA	58585070	406067	Royal Jelly	CG1629
GB16324-PA	67010041	409873	Royal Jelly	CG1629
GB12951-PC	48097086	408289	Signalling	CG17870
GB15202-PA	66531474	551882	Signalling	CG1349
GB15582-PA	48096523	408951	Signalling	CG31196
GB16178-PA	66520994	413211	Signalling	CG5811
GB16716-PA	110748765	725041	Signalling	CG8561
GB16072-PA	66550870	409485	Signalling/AntiOx	CG6342
GB10536-PA	94158822		Small molecular binding protein	-
GB19662-PA	110766389	727028	Small molecular binding protein	CG2016
GB19745-PA	58585086	406078	Small molecular binding protein	CG6186
GB10009-PA	66524874	412886	Structural	CG1913
GB10091-PA	48132776	413256	Structural	CG7802
GB10122-PA	110762983	410559	Structural	CG9277
GB10275-PA	48095525	408782	Structural	CG9277
GB10514-PA	66521545	408388	Structural	CG1913
GB11282-PA	66512737	412799	Structural	CG1071
GB11920-PA	48095543	410994	Structural	CG3401
GB12614-PA	66509769	410075	Structural	CG18290
GB13049-PA	48095547	410996	Structural	CG9277
GB13229-PA	110760290	410204	Structural	CG30084
GB13999-PA	58585104	406088	Structural	CG31150
GB15794-PA	66546405	410975	Structural	CG7802
GB16448-PA	110766532	409533	Structural	CG5730
GB17673-PA	110762380	408396	Structural	CG6831
GB17681-PA	48137684	406122	Structural	CG4027
GB18917-PA	110751158	725718	Structural	CG4254
GB12113-PA	66521459	551325	Transport	CG6647
GB14012-PA	66525867	413517	Transport	CG9090
GB16577-PA	110755759	411052	Transport	CG3036
GB11987-PA	110764400	726641	Unknown	-
GB12562-PA	66522463	408421	Unknown	CG10513
GB13778-PA	110755198	551170	Unknown	CG33196
GB14970-PA	110749723	409731	Unknown	CG18251
GB15662-PA	110749015	724721	Unknown	CG10962
GB17311-PA	110766503	552228	Unknown	CG8444
GB17500-PA	110755329	725960	Unknown	-
GB19255-PA	48098542	408538	Unknown	CG1155
GB30569-PA	AmeLG8_WGA346_4 [LG8]		Unknown	-

Details of the PreRelease 2 accession numbers, the NCBI RefSeq GI numbers and the Bee Gene ID numbers are provided in the first three columns. Functional groups and corresponding genes in *Drosophila *(Additional data file 4) are provided in separate columns for each protein identified in the spermathecal fluid of the honeybee.

**Figure 3:**
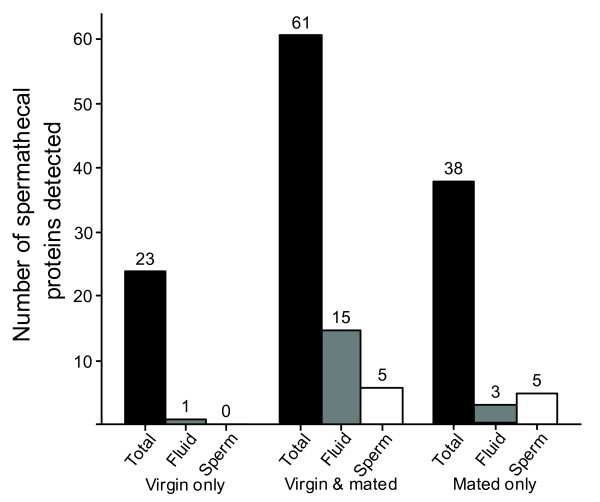
Spermathecal fluid proteins in virgin and mated queens. A graphical comparison of the spermathecal proteins detected in our study. The black bars show the total number of proteins that were detected in both virgin and mated queens as well as the number of proteins detected in virgins or mated queens only. The number of spermathecal proteins that were also found in seminal fluid and sperm are shown by grey and white bars, respectively. Half of the spermathecal proteins (50%) were found in mated as well as virgin queens, although subsets of proteins were unique for mated (30%) and virgin queens (20%). Overlaps of spermathecal proteins with those reported for sperm and seminal fluid [19] were generally low and are shown by the grey and white bars, respectively.

We compared our list of 122 spermathecal proteins with the reported abundant proteins from bee sperm samples [19]; we found that only 10 (8%) proteins were detected in both the spermathecal fluid and this list of sperm proteins (Figure 3; Additional data file 1). We also detected five of these ten sperm proteins in the spermathecal fluid of virgin queens, so it is unlikely that these are contaminating sperm proteins but instead represent the expression of the same gene that queens secrete into the spermathecal fluid. Only 5 (4%) proteins were found in sperm samples in our previous publication from male ejaculates and also in the spermathecal fluid list from mated queens presented here (Figure 3). Comparison of the spermathecal list with the top 12 most abundant hemolymph proteins we have previously detected by mass spectrometry [19] also revealed no overlap. We have also compared the protein profiles of spermathecal fluid identified here and our previous analysis of seminal fluid [19] and again found substantial differences. Only 19 (16%) out of the set of 122 spermathecal proteins were also detected in this previously reported seminal fluid proteome. Sixteen of this set of 19 proteins were also present in the spermathecal fluid of virgin queens and, thus, cannot be considered as contaminants from male seminal fluid (Table 1; Additional data file 1). This provides evidence that while qualitative assessment of seminal fluid contamination in our spermathecal fluid samples was minimal at the depth of the analysis performed, some identical proteins are present, which appear to be expressed and secreted by both males into their ejaculate and by females into the spermatheca. Our dataset of 122 proteins also allowed a comparison of the spermathecal protein population of virgin and mated queens. We detected peptides for 61 proteins present in both virgin and mated queens (Figure 3), but each group also had unique sets of proteins not found in the other. We found that 38 (30%) spermathecal fluid proteins were only detected in mated queens and 23 (19%) proteins were only detected in virgin queens. Obviously, protein profiles differ between young, virgin and old inseminated queens, but our study was not able to distinguish whether this proteomic changes are caused by queen age or mating status. Future work will be needed to resolve this issue; however, aged virgin females are physiologically and technically extremely difficult to obtain to test this issue.

Spectral counts in our LC-MS/MS data from spermathecal fluid revealed that counts for particular proteins were sometimes substantially different between mated and virgin queens (Additional data file 2). This indicates that the protein concentrations might substantially differ between spermathecal fluid of mated and virgin queens. Future work is obviously needed to quantify the proteins with different spectral counts. To do this, biological replicates of spectral counts based on LC-MS/MS will be necessary, but were beyond the scope of the current study.

**Figure 4:**
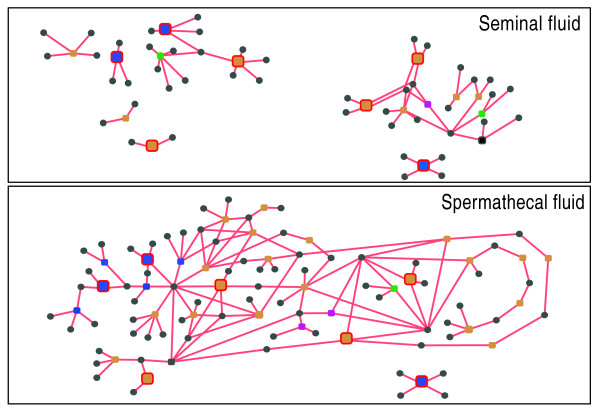
Metabolic networks of seminal and spermatecal fluid. Visualization of spermathecal and seminal fluid metabolic networks based on the proteins identified in this study and Baer *et al*. [19]. Colored nodes (rounded squares) represent enzymes in different functional categories, metabolites are shown as small grey circles, and reactions are shown as connecting lines between the enzyme and metabolite nodes. Additional data file 3 provides Enzyme Commission (EC) numbers and metabolite names for all features that are noted. The seven enzymes in common between the two datasets are highlighted by increased size, and red outlines indicate nodes with a consistent spatial arrangement in both networks.

To further explore the metabolic network established in the spermathecal fluid, we created metabolic networks of spermathecal fluid and seminal fluid using data from the Kyoto Encyclopedia of Genes and Genomes (KEGG) [36,37] associated with our identified proteins. This was then visualized with the Cytoscape software package [38]. The resulting networks are presented in Figure 4 (see also an annotated version provided as Additional data file 3), where colored nodes (rounded squares) represent enzymes in different functional categories, metabolites are shown as small grey circles, while the reaction is shown as connecting lines between the enzymes and metabolite nodes. The two networks differ in their degree of connectivity and the number of hubs that join multiple reactions. In the seminal fluid network there are discrete metabolic reactions leading to six clusters of reactions plus the redox reaction of disulfide isomerase. This is consistent with sperm needing only to survive for a short period in seminal fluid and the substrates necessary for these reactions being pre-charged in seminal fluid prior to ejaculation. In contrast, the spermathecal fluid is a well-connected single metabolic entity. It contains 5 of the 14 enzyme nodes present in the seminal fluid, but also an extra 23 enzyme nodes that combine the 6 clusters in the seminal fluid into a single metabolic network. Obviously, the different metabolic steps are interlinked with many products representing the substrates for other reactions. This correlates with the requirement of spermathecal fluid to maintain homeostatic functions for years, perhaps with only a small set of entry metabolites. The terminal metabolite nodes of the network are potential substrates to be transported in or out of the spermatheca, across the spermathecal wall.

The spermathecal network shows the key features of biochemistry needed for sperm protection and maintenance. It shows a near complete glycolytic pathway that is absent from the seminal fluid and a large series of components for a vacuolar like protein pumping ATPase. It also contains a variety of antioxidant defenses, most shared with seminal fluid proteins, although it is often different gene products that catalyze the same reactions. These three networks interact through common ATP/ADP and NAD(P)/NAD(P)H pools (Additional data file 3).

Protons (H^+^) are presented as metabolites here and are heavily connected nodes (Additional data file 3); we kept these in the network given that metabolic maintenance of pH may be an important function in spermathecal fluid [39]. However, removal of this 'currency metabolite' [40] does not significantly break the highly interconnected structure of the spermathecal fluid network, but it does further fragment the seminal fluid network (data not shown).

## Discussion

The first large-scale identification of proteins that are present in the spermathecal fluid of honeybee queens is an essential step in uncovering the molecular regulation of long-term sperm storage. A comparison of identified protein lists between our spermathecal fluid samples and those from sperm and hemolymph revealed surprisingly little overlap. Our analysis of spermathecal fluid of virgin queens, which could not have been contaminated with sperm proteins, allowed us to further decrease the number of possible sperm contaminants to only five proteins that we subsequently removed from our final list to avoid any form of contamination from stored sperm. The detection of these remaining sperm proteins in spermathecal fluid does not necessarily result from contamination, as proteins might be expressed in both locations *in vivo*. Information about the proteineous contributions of females towards stored sperm is still very limited. An expressed sequence tag analysis in *Drosophila *detected 42 transcripts that are enriched for expression in the spermatheca [41] but we noted that only 3 proteins within the honeybee spermathecal proteome list had significant sequence similarity to proteins predicted from these *Drosophila *transcripts. A set of 19 genes highly expressed in spermathecae were identified during analysis of the *Hr39 *gene in *Drosophila*, which is reported to regulate *Drosophila *female reproductive tract development and function [42]. While there are orthologs for most of these proteins in *Apis*, only one of the *Drosophila *genes highly expressed in spermathecae (*Hsc70-4*) has orthologs in our protein set. These orthologs are among the heat shock protein molecular chaperones (Table 2). Recently released microarray analysis of virgin and mated spermatheca from *Drosophila *[43,44] reveals a large number of spermatheca enriched transcripts. Sequence comparison with the *Apis *spermathecal proteins in Table 2 reveal that approximately 47% of the corresponding genes in *Drosophila *have significant spermatheca-enriched expression patterns, while a further 30% have significantly spermatheca-depleted expression patterns (Additional data file 4).

The spermathecal fluid proteins of the honeybee differ substantially from those we have reported in seminal fluid [19], supporting the idea that selection on seminal and spermathecal fluid were substantially different. Seminal fluid was selected to increase insemination and paternity success whereas spermathecal fluid evolved to maximize sperm survival. Nevertheless, we were surprised by the finding of a small 20% overlap between these two protein sets (Figure 2 and Table 1) given that seminal fluid and spermathecal fluid are expected to also share common roles, such as keeping sperm alive, reducing oxidative stress, nourishing sperm or protecting sperm from microbial attacks. The network analysis shows that while different proteins are involved, many biochemical classes and enzymatic functions are the same in both fluids. Indeed, previous research in ants [6] and honeybees [7] shows that both spermathecal fluid and seminal fluid keep sperm viable, but we here show that the specific proteins to achieve this differ substantially between the male and female. Sperm is obviously able to survive in both of these 'habitats' but it might have to undergo developmental changes at the beginning of its storage to achieve this. Our finding that spermathecal fluid of virgins, which are anticipating freshly ejaculated sperm to arrive in the spermatheca, differs, in part, from that in mated queens (Figure 3), where sperm has been stored for several months, supports this idea. Consequently, the sperm storage process might be more complicated than assumed so far, and may involve a period of adjustment when the female partially mimics the seminal fluid environment but then modifies the conditions. This may minimize the energetic costs of sperm storage over time or select for specific sperm traits and thereby manipulates the paternity success of her mates.

Some of the components of the spermathecal fluid are likely linked to the need for protection of the sperm from damaging infections or damaging chemical substances that might be detrimental to long term storage. For example, several chitinases were found that might be used in defense for degrading fungal cell walls [RefSeq Gi 66514614, 110760993, 66511507]. We also found an elaborate antioxidant defense system of nine different enzymes, including defenses against superoxide, hydrogen peroxide and lipid peroxides, that likely help prevent oxidative damage to sperm during their substantial hiatus. This is consistent with the evidence of a high activity of several antioxidant defense enzymes in spermathecal fluid [8]. Also, we found a number of chelating proteins, several with roles in Fe^2+ ^binding, which again may represent an antioxidant defense by preventing metal-catalyzed reactive oxygen species production and/or a scavenging of metals to prevent their use in the growth and proliferation of bacterial or fungal infections.

The most prominent aspect of the spermathecal metabolic network is glycolytic metabolism, which is a pathway for fructose degradation to organic acids and the production of both NADH and ATP (Figure 4). NADH will be needed to fuel the antioxidant enzymes noted above. ATP from this extracellular glycolysis could be used to fuel the vacuolar-like ATPase (Table 1). In many animal cell types, such an ATPase normally hydrolyzes cellular ATP and is used to pump protons out of cells, leading to raising of cellular pH and activation of K^+ ^influx channels that replace the expelled H^+ ^with K^+ ^[45,46]. The long established basic pH and high K^+ ^concentration in the spermatheca that has been hypothesized to slow sperm metabolic rate [47,48] could be catalyzed by such an ATPase pump activity. However, to our knowledge, such pumps have not been reported to operate in the direction required here, raising extracellular pH, so the link between vacuolar-like ATPases and the spermathecal pH and K^+ ^concentrations requires more research.

An intriguing possibility is that this glycolytic pathway is also feeding carbon substrates to the sperm to maintain their own internal metabolism. Fructose as a carbon source seems to be of specific importance for honeybees [49] and the dominance of gycolytic pathway proteins in male reproductive organs has been reported earlier [20]. Klenk *et al*. [5] previously identified the glycolytic enzyme triosephosphate isomerase as a mating enhanced component of the honeybee spermathecal fluid. Together, our evidence is significant for an extracellular glycolytic pathway operating in the spermathecal fluid. This could suggest a change in primary carbon substrate for sperm, because in seminal fluid they are fueled by their own internal energy stores. This switch in substrates may be critical in establishing a new, slower metabolic rate required for long-term homeostasis in the spermatheca.

## Conclusions

Our large-scale identification of proteins within the spermathecal fluid of honeybee queens offers an intriguing insight into the details of female sperm storage. Our data indicate that females provide stored sperm with a complex mixture of proteins that form a metabolically connected network. They also suggest that some essential physiological requirements of sperm have effectively been 'outsourced' and are now provided by the female. In this respect, sperm storage could be regarded as a specialized from of endosymbiosis between males and females, post-copulation but pre-fertilization.

## Materials and methods

### Sample preparation

Spermathecal fluid was collected by dissecting virgin and mated queens using a Leica stereo microscope at 40× to 62× magnification. All dissections were performed with fine watchmaker forceps (INOX 5, Biology) and in Hayes solution (9.0 g/l NaCl, 0.2 g/l CaCl_2_, 0.2 g/l KCl, 0.1 g/l NaHCO_3_, pH 8.7). Spermathecal fluid was sampled from a total of 206 mated and 64 virgin queens. Mated queens were egg laying mother queens at least 9 months of age and were provided by several local beekeepers. Virgin queens were obtained by grafting and used at an average age of 6 days, being the age when queens typically perform their nuptial flights. To sample spermathecal fluid, queens were briefly anesthetized in CO_2 _for 20 to 30 seconds after which their spermathecae were immediately dissected and transferred to a drop of Hayes solution. The dense tracheal network surrounding the spermatheca was carefully removed. The spermatheca was then washed in a second drop of Hayes to minimize contamination by hemolymph. The spermatheca was then placed on a microscopic slide. After the removal of remaining Hayes an injection needle was used to pierce a small whole into the spermathecal wall. The spermathecal fluid was then collected out of the lumen using a fine glass capillary. For each biological sample we pooled samples from 20 to 30 queens. For the samples from mated queens spermathecal fluid was separated from the surrounding stored sperm by centrifugation for 25 minutes at 850 × g at 4°C. The supernatant (spermathecal fluid) was collected and centrifuged again at 18,620 × g for 10 minutes at 4°C to remove remaining sperm. Samples from virgin queens were briefly centrifuged at 10,000 × g but not processed any further and all spermathecal fluid samples were frozen at -80°C prior to further analyses. To collect secretions of the spermathecal glands, we collected up to 20 glands for each biological sample and kept them in 50 μl of Hayes on ice. The glands were then carefully opened at their distal end using watchmaker forceps to allow the gland content to dissolve into the surrounding solution. Separation of the gland tissue from the dissolved gland secretions was done by centrifugation for 20 minutes at 850 × g and at 4°C.

### Protein profiling using gel electrophoresis

Profiling of spermathecal fluid proteins was performed by SDS-PAGE using either Biorad Criterion precast gels (10 to 20% (w/v) acrylamide, HCl, 1 mm, 18 comb) or larger 12% (w/v) acrylamide homemade slab gels (Hercules, CA, USA). Gels were run at 30 mA, fixed in fixing solution (40% methanol, 10% acetic acid) for an hour and stained overnight with colloidal Coomassie blue (G 250). Gels were kept in 0.5% (v/v) phosphoric acid at 4°C prior to protein identifications using peptide mass spectrometry.

### Identification of proteins from gels using tandem mass spectrometry

Colloidal Coomassie blue stained protein spots were cut from gels and destained twice in 10 mM Na_2_HCO_3 _with 50% (v/v) acetonitrile. Samples where dried at 50°C before being rehydrated with 15 μl of digestion solution (10 mM NH_4_CO_3 _with 12.5 μg/ml trypsin (Invitrogen, Carlsbad, CA, USA) and 0.01% (v/v) trifluoroacetic acid) and incubated over night at 37°C. Peptides produced from trypsinization were twice extracted from gel plugs using 15 μl acetonitrile. The supernatant was then collected and plugs washed twice with 15 μl of 50% (v/v) acetonitrile and 5% (v/v) formic acid and combined with initial supernatant. The pooled extracts were dried by vacuum centrifugation and stored at 4°C before being analyzed by mass spectrometry.

#### Gel spot protein identifications

Samples from excised gel pieces were analyzed on an Agilent XCT Ultra IonTrap mass spectrometer with an electrospray ionization (ESI) source equipped with a low flow nebuliser in positive mode and controlled by Chemstation (rev. B.01.03 [204]; Agilent Technologies, Santa Clara, CA, USA) and MSD Trap Control software version 6.1 (Bruker Daltonik GmbH, Bremen, Germany). Peptides were eluted from a self-packed Microsorb (Varian Inc., Palo Alto, CA, USA) C18 (5 μm, 100 Å) reverse phase column (0.5 × 50 mm) using an Agilent Technologies 1100 series capillary liquid chromatography system at 10 μl/minute using a 9 minute acetonitrile gradient (5 to 60% (v/v)) in 0.1% (v/v) formic acid at a regulated temperature of 50°C. The method used for initial ion detection utilized a mass range of 200 to 1,400 m/z with scan mode set to 'standard' (8,100 m/z per second) and ion charge control conditions set at 250,000 and 3 averages taken per scan. Smart mode parameter settings were employed using a target of 800 m/z, a compound stability factor of 90%, a trap drive level of 80% and optimize set to 'normal'. Ions were selected for MS/MS after reaching an intensity of 80,000 cps and two precursor ions were selected from the initial mass spectrometry scan. MS/MS conditions employed SmartFrag for ion fragmentation, a scan range of 70 to 2,200 m/z using an average of 3 scans, the exclusion of singly charged ions option and ion charge control conditions set to 200,000 in Ultra scan mode (26,000 m/z per second). Resulting MS/MS spectra were exported from the DataAnalysis for LC/MSD Trap version 3.3 (build 149) software package (Bruker Daltonik GmbH) using default parameters for AutoMS(n) and compound 'export'. The resulting .mgf files were then searched as outlined below.

#### Whole lysate protein identifications

Spermathecal fluid proteins of mated as well as virgin queens were also analyzed with a non-gel approach, using complex mixture LC-MS/MS analysis. Spermathecal samples were digested overnight at 37°C with trypsin and insoluble components were removed by centrifugation at 20,000 × g for 10 minutes. Samples were analyzed on an Agilent 6510 triple quadrupole mass spectrometer (Q-TOF) mass spectrometer with an HPLC Chip Cube source. The chip consisted of a 40 nl enrichment column (Zorbax 300SB-C18 5 u) and a 150 mm separation column (Zorbax 300SB-C18 5 u) driven by Agilent Technologies 1100 series nano/capillary liquid chromatography system. Both systems were controlled by MassHunter Workstation Data Acquisition for Q-TOF (version B.01.02, build 65.4, Patches 1,2,3,4; Agilent Technologies). Peptides were loaded onto the trapping column at 4 μl min^-1 ^in 5% (v/v) acetonitrile and 0.1% (v/v) formic acid with the chip switched to enrichment and using the capillary pump. The chip was then switched to separation and peptides eluted during a 1 h gradient (5% acetonitrile to 40% acetonitrile) directly into the mass spectrometer. The mass spectrometer was run in positive ion mode and scans run over a range of 275 to 1,500 m/z and at 4 spectra s^-1^. Precursor ions were selected for auto MS/MS at an absolute threshold of 500 and a relative threshold of 0.01, with a maximum of 3 precursors per cycle, and active exclusion set at 2 spectra and released after 1 minute. Precursor charge-state selection and preference was set to 2+ and then 3+ and precursors selected by charge then abundance. Resulting MS/MS spectra were opened in MassHunter Workstation Qualitative Analysis (version B.01.02, build 1.2.122.1, Patches 3; Agilent Technologies) and MS/MS compounds detected by 'Find Auto MS/MS' using default settings. The resulting compounds were then exported as mzdata files that were then searched as outlined below.

#### Database searching

Mass spectra output files were analyzed against the predicted *A. mellifera *peptide set (PreRelease2, 11,069 sequences; 5,989,390 residues) from BeeBase [50] using the Mascot search engine version 2.2.03 (Matrix Science, Boston, MA, USA). Gel spot searches were conducted using the Mascot search engine version 2.2.03 (Matrix Science) utilizing error tolerances of ± 1.2 Da for MS and ± 0.6 Da for MS/MS, 'Max missed cleavages' set to 1, the Oxidation (M) variable modifications and the instrument set to ESI-TRAP and peptide charge set at '2+ and 3+'. Results were filtered using 'Standard scoring', 'Max. number of hits' set to 20, 'Significance threshold' at *P *< 0.05. Complex lysate searches were conducted using the Mascot search engine version 2.2.03 (Matrix Science) utilizing error tolerances of ± 100 ppm for MS and ± 0.5 Da for MS/MS, 'Max missed cleavages' set to 1, the Oxidation (M) variable modifications and the instrument set to ESI-Q-TOF and peptide charge set at 2+ and 3+. Results were filtered using 'MUDPIT scoring', 'Max. number of hits' set to 20, 'Significance threshold' at *P *< 0.05. Lists of the spermathecal fluid protein sets identified for the various samples and scores for matches are provided as Additional data file 1. To build the protein list, we applied conservative approaches to minimize false positives. Protein matches were only claimed if at least two distinct peptides were detected per protein, and MOWSE (molecular weight search) scores being higher than 50 (*P *< 0.05 significance level is a score >37). False discovery rate analysis of the trypsin digested spermathecal fluid samples from virgin and mated queens against a decoy randomized *A. mellifera *protein set (PreRelease2, 11,069 sequences; 5,989,390 residues) revealed a <2.5% false discovery rate for the virgin queen sample and a <2.5% false discovery rate for the sample from mated queens.

Each protein sequence identified from the *Apis *protein set was submitted to a BLAST search to identify homologous proteins from insects and other organisms. This process was used to confirm or modify the functional annotation of the proteins from the PreRelease2 dataset, and then each protein was placed into a functional category according to its annotation and manual literature searches where necessary.

### Network analysis and visualization

From the KEGG database [36,37] of biochemical pathways, proteins identified in the present study (Table 1) and [19] were associated with unique ID 'dame' entries specific to *A. mellifera *enzymes. Following this step, enzyme commission (EC) numbers, enzyme names and reactions associated with these KEGG IDs, where these exist, were extracted with a Perl script from the 'enzyme' file downloaded from the KEGG ftp site [51]. Proteins for which no EC number could be assigned typically have unknown function or are responsible for non-enzymatic processes. A total of 41 of the honey bee proteins (using the PreRelease2 accession numbers) in our spermathecal set shown in Table 1 were assigned EC numbers in this manner, making a non-redundant set of 33 enzyme nodes and 70 metabolites. Similarly, seminal fluid proteins from [19] yielded a non-redundant set of 16 enzyme nodes and 47 metabolites.

After the recovery of these data, the set of unique EC numbers and biochemical reactions was parsed to generate a simple interaction format (SIF) file to represent a metabolic network. The SIF file and other data, such as GB codes associated with EC numbers, enzyme names, and node types (enzyme or metabolite), were inputted into the Cytoscape software (version 2.6.0) [32] for network visualization and analysis. Network images were exported from Cytoscape as .svg files, imported into Adobe Illustrator and modified visually for presentation purposes.

## Abbreviations

EC: Enyme Commission; ESI: electrospray ionization; KEGG: Kyoto Encyclopedia of Genes and Genomes; LC: liquid chromatography; MOWSE: molecular weight search; MS: mass spectrometry; MS/MS: tandem mass spectrometry; NCBI: National Center for Biotechnology Information; Q-TOF: triple quadrupole mass spectrometer.

## Authors' contributions

BB carried out the experimental work, analyzed the data and wrote the paper; HE participated in the SDS PAGE work and MS/MS; NLT performed all MS/MS runs, NOT performed the network analysis and AHM analyzed the data and co-wrote the paper. All authors read and approved the final manuscript.

## Additional data files

The following additional data are available with the online version of this paper: a table showing identification of proteins in honeybee spermathecal fluid by MS/MS analysis of two one-dimensional gels and two gel-free analyses of tryptic peptides (Additional data file 1); a table listing peptide counts from mated and virgin spermathecal fluid using tandem MS analysis of gel-free analyses of tryptic peptides (Additional data file 2); a figure illustrating the metabolic network of seminal and spermatecal fluid (Additional data file 3); a table listing abundances of *Drosophila *transcripts with sequence similarity to the proteins found in *Apis *spermatheca (Additional data file 4).

## Supplementary Material

Additional data file 1 MS/MS spectra derived from trypsinated peptides of spermathecal fluid proteins were matched using Mascot (Matrix Sciences) against Honey Bee PreRelease 2.0. In each case: 'MOWSE' is the Mascot reported molecular weight search score (>37 is *P *< 0.05); 'Peptides' is the number of peptides matched to the protein above the threshold as outlined in materials and methods; and 'Gelspot' refers to the protein band number as shown in Figure 1. Shading indicates multiple matching data to the same *Apis *predicted protein sequence from gel bands. Seminal fluid and sperm data on the same proteins are reproduced from [19]. Bee genome GB number and the corresponding RefSeq protein GI from NCBI are given along with the assembly 4.0 gene ID for the bee genome. Click here for file

Additional data file 2 MS/MS spectra derived from trypsinated peptides of spermathecal fluid proteins were matched using Mascot (Matrix Sciences) against Honey Bee PreRelease 2.0. In each case 'Spectra' is the number of spectra matching to each protein as outlined in Materials and methods. Bee genome GB number and the corresponding RefSeq protein GI from NCBI are given. Click here for file

Additional data file 3 Visualization of spermathecal and seminal fluid metabolic networks based on the proteins identified in this study and Baer *et al*. [19]. Colored nodes (rounded squares) represent enzymes in different functional categories, metabolites are shown as small grey circles, and reactions are shown as connecting lines between the enzyme and metabolite nodes. EC numbers are listed near enzyme nodes and metabolite names for all features are noted. The seven enzymes in common between the two datasets are highlighted by increased size, red outlines and consistent spatial arrangement in both networks. Click here for file

Additional data file 4 Abundances are fluorescence signals from microarrays of virgin and mated spermatheca compared to the average signal in the whole fly average data. Data are derived from [41]. Click here for file
